# LncRNA GAS5 represses stemness and malignancy of gliomas *via* elevating the SPACA6-miR-125a/let-7e Axis

**DOI:** 10.3389/fonc.2022.803652

**Published:** 2022-08-29

**Authors:** Shuang Wu, Kaixi Ren, Jing Zhao, Juan Li, Bo Jia, Xiuquan Wu, Yanan Dou, Xiaowei Fei, Yu Huan, Xin He, Tingting Wang, Weihao Lv, Li Wang, Yan’gang Wang, Junlong Zhao, Zhou Fei, Sanzhong Li

**Affiliations:** ^1^ Department of Neurosurgery, Xijing Hospital, Air Force Military Medical University, Xi’an, China; ^2^ Department of Neurology, Tangdu Hospital, Air Force Military Medical University, Xi’an, China; ^3^ Department of Anesthesiology, Xijing Hospital, Air Force Military Medical University, Xi’an, China; ^4^ State Key Laboratory of Cancer Biology, Department of Medical Genetics and Developmental Biology, Air Force Military Medical University, Xi’an, China

**Keywords:** Glioblastoma (GBM), growth arrest-specific transcript 5 (GAS5), stem cell, miR-125a, let-7e, IL6, STAT3.

## Abstract

Glioblastoma (GBM) is a highly invasive neurological malignancy with poor prognosis. LncRNA-GAS5 (growth arrest-specific transcript 5) is a tumor suppressor involved in multiple cancers. In this study, we explored the clinical significance, biological function, and underlying mechanisms of GAS5 in GBM. We showed that lncRNA-GAS5 expression decreased in high-grade glioma tissues and cells, which might be associated with poor prognosis. GAS5 overexpression lowered cell viability, suppressed GBM cell migration and invasion, and impaired the stemness and proliferation of glioma stem cells (GSCs). We further discovered that GAS5 inhibited the viability of glioma cells through miR-let-7e and miR-125a by protecting SPACA6 from degradation. Moreover, GAS5 played an anti-oncogenic role in GBM through the combined involvement of let-7e and miR-125a *in vivo* and *in vitro*. Notably, these two miRNAs block the IL-6/STAT3 pathway in tumor tissues extracted from a xenograft model. Taken together, our study provides evidence for an important role of GAS5 in GBM by affecting the proliferation and migration of GSCs, thus providing a new potential prognostic biomarker and treatment strategy for GBM.

## Introduction

Glioblastoma (GBM) is the most common malignant primary brain tumor in adults, with a median survival of barely 1.3 years upon detection (1, 2). The discouraging overall prognosis of gliomas has changed little over the decades despite medical advances in neurosurgery, chemotherapy, radiation, and many novel clinical trials (3). The proliferation and invasion capacities of GBMs make it extremely difficult for tumors to be resected completely. With the advent of molecular biology, new studies on gene regulation networks have provided directions for understanding the molecular mechanisms of intracranial tumors (4, 5). The classification of gliomas was reorganized by the 2016 revision of the World Health Organization Classification of Tumors of the Central Nervous System (CNS), which highlights molecular features in addition to histopathological appearance (6). Notably, the malignant features of glioma cells, including invasive growth, resistance to conventional chemotherapy or radiotherapy, and recurrence, are mainly attributed to glioma stem cells (GSCs). The GSCs display three prominent features of stem cells, i.e. self-renewal capacity, indefinite proliferation, and differentiation (7). Hence, GSCs are responsible for not only the infinite growth of GBM but also incomplete and residual tumor resection, which generally results in a poor prognosis after treatment. Therefore, a comprehensive understanding of the molecular pathogenesis of GSCs is urgently required.

Noncoding RNAs (ncRNAs) consist of microRNAs (miRNAs) and long ncRNAs (lncRNAs). LncRNAs are a type of RNAs of more than 200 nucleotides (nt) in length, and there is no solid evidence to prove that they encode peptides (8). Numerous studies have shown that lncRNAs play crucial roles in the malignant biology of tumors. Abnormal expression of lncRNAs in tumors indicates tumor progression as a specific marker for certain tumors and is likely to become a new therapeutic target (8, 9). It has been shown that lncRNAs are involved in chromosome remodeling or gene expression *via* post-transcriptional regulation (10–12), alternative splicing (13), and initiated X-chromosome inactivation (14). As suggested in recent studies, lncRNAs may influence cell proliferation, migration, and invasion *in vitro* and participate in the DNA damage response, tumorigenesis, and drug resistance (15–17). miRNAs are transcripts of approximately 21-23 nucleotides in size that are produced in the nucleus and cytoplasm of cells through a multi-step process (18). Traditionally, some abundant lncRNAs with complementary miRNA loci can act as competitive endogenous RNAs or miRNA sponges through direct base pairing to regulate gene expression, thereby inhibiting the function of miRNA-targeted mRNAs (19). Accumulating evidence has shown that lncRNAs are related to the stemness of GSCs through epigenetic inhibition of genes associated with multi-neuron differentiation or impact GSCs proliferation, migration, and invasion by binding to certain miRNAs and regulating downstream molecular pathways (20–22). Therefore, elucidation of the mechanisms and underlying relationship between lncRNAs and GSCs will help offer a new focus on the tumorigenesis and therapeutic response of GBM.

In recent years, LncRNA-GAS5 (growth arrest-specific transcript 5) has been reported as a tumor suppressor lncRNA in various human cancers. For instance, its downregulation has been observed in epithelial ovarian cancer, cervical cancer, lung cancer, and gliomas (23–27). GAS5 directly interacts with the WW structural domain of YAP to inhibit colorectal cancer progression *in vitro* and *in vivo* (28). Wang et al. found that GAS5 overexpression inhibits M2-like polarization of tumor-associated macrophages by enhancing the expression of PTEN, thereby inhibiting the proliferation and invasion of liver cancer cells (29). In addition, Ding et al. suggested that GAS5 attenuates glioma progression by eliminating microRNA-10b and Sirtuin1 (30). Therefore, new insights into the molecular mechanisms underlying GAS5 will provide important directions for future research. In this study, we identified a remarkable decrease in GAS5 expression in glioma tissues and cells, which was closely related to the viability and migration of GBM cells and GSCs. SPACA6 and its downstream miRNAs, let-7e, and miR-125a played a role in these phenomena, and the IL-6/IL-6R/STAT3 pathway is involved in the molecular mechanism of GAS5 as an anti-oncogene. These results indicate that GAS5 could attenuate GSCs activity and suppress glioma progression, which may provide novel strategies for glioma therapy.

## Materials and methods

### Ethics statement

The research strategy was approved by the Research Ethics Committee of the Xijing Hospital of the Airforce Medical University. All experiments were performed in accordance with relevant guidelines and regulations, and written informed consent was obtained from all the patients involved. Written consent for the use of all data was also obtained from the lead researcher (Principle Investigator, PI) of the neurosurgical glioma center of Xijing Hospital. The privacy rights of the human subjects were observed in our study.

### Clinical specimens

Glioma tissues were obtained from 72 patients who underwent primary surgery and were histopathologically diagnosed with glioma at the Xijing Hospital. Samples were collected and stored in liquid nitrogen, and the remaining clinical data were obtained from the hospital case system and follow-up of patients.

### Cell lines and cell culture

Four human GBM cell lines (U251, U87, A172, and T98) and a healthy glial cell line (HEB) were obtained from the American Type Culture Collection (ATCC), while other human GBM cell lines (SHG44) were purchased from the Wuhan Procell Life Technology Collection. The cells were cultured in DMEM (Gibco, USA) medium supplemented with 10% fetal bovine serum (FBS, Hyclone, USA) at 37 °C in a humidified environment with 5% CO_2_. The cells tested negative for mycoplasma contamination. Patient-derived glioblastoma stem-like cells (GSC-1 and GSC-2) were isolated from surgical samples. Briefly, tissues were mechanically minced and then digested with 0.1% trypsin (Invitrogen, USA) and 10 U mL^−1^ of DNase I (Promega, USA) at 37 °C for 45 min. ACK lysis buffer (Beyotime, China) was used to lyse red blood cells. The washed tissues were triturated by pipetting, passed through a 40 μm cell strainer, and grown in DMEM/F12 (Gibco, USA) supplemented with B27 (Gibco, USA), EGF (20 ng/mL) (Propotech, USA), bFGF (20 ng/mL) (Propotech, USA), and 1% penicillin/streptomycin (Life Technologies, China).

### RNA extraction and quantitative real-time polymerase chain reaction (qRT-PCR)

Total RNA was extracted from GBM tissues and cancer cells using the TRIzol reagent (Invitrogen, USA). The concentration and purity of RNA were measured using an Eppendorf Biophotometer Plus. The PrimeScript™ RT Reagent Kit (Takara, Japan) was used to reverse-transcribe the lncRNAs and mRNA. Relative gene expression levels were determined using a SYBR Green PCR Kit (Takara, Japan). The relative quantity of gene expression was calculated using the standard 2^−ΔΔCt^ method, with GAPDH as an internal control. Primer sequences are listed in **Supplementary Table 1**.

### Cell counting kit-8 (CCK-8) assay

Cells were seeded in 96-well plates at a density of 2×10^3^ cells/well, and cell viability was assessed using the CCK-8 Assay Kit (Dojindo Molecular Technologies, Japan), as instructed by the manufacturer. Samples were measured at OD 490 nm using the Bio-Rad Microplate Reader Model 680 (Bio-Rad, China).

### Colony formation experiment

The colony formation experiment was performed 48 h after transfection. The cell lines were resuspended and plated in 10 cm culture plates at a densities of approximately 50/100/200 cells/well. The cells were cultured under standard conditions for three weeks, and the culture medium was changed every three days. Colonies formed by the clones were collected, washed twice with phosphate-buffered saline (PBS), fixed with formaldehyde solution, and stained with Giemsa stain (Xi ‘anYike Biotechnology Co., LTD, China). Colony formation was observed and photographed using an optical microscope and a digital camera.

### Flow cytometry

CD133 expression and cell apoptosis were measured by flow cytometry. To detect the expression of CD133, a single-cell suspension was obtained from well-grown attached cells by trypsinization (without EDTA) under sterile conditions. Samples were stained with IgG1 Isotype Control Summary (R&D Systems, USA) for approximately 15 min at 4°Cin the dark. Next, cells were washed with cold PBS, centrifuged at 1000 rpm for 5 min at 4 °C, and subsequently stained with the human CD133-PE antibody (eBioscience, Thermo Scientific, USA) for approximately 30 min at 4°Cin the dark. A fluorescence-activated cell sorting (FACS) instrument, CantoII Flow Cytometer (BD Biosciences, USA), was used to examine the results.

Cells were plated in 6-well plates for the detection of apoptosis. After transfection, a single-cell suspension was obtained, and the cells were stained using the Annexin V-Fluorescein Isothiocyanate (FITC) Apoptosis Detection Kit (Beyotime, China) for 15 min at room temperature. Finally, the analysis was conducted using a FACS CantoII Flow Cytometer (BD Biosciences, USA) and FlowJo 10.0 software.

### Scratch test

Cells were seeded in 6-well plates at a density of 5×10^5^ cells/well. After transfection, the cells with complete confluence were allowed to draw five uniform straight lines along the central axis at a right angle to the bottom of the 6-well plate using a sterile 10-μL pipette tip. After rinsing three times with PBS, the cells were cultured in serum-free medium for 0.5 h. Subsequently, cell migration was observed and photographed at 0, 24, and 48 h under an inverted optical microscope.

### Transwell assay

The transwell assay was performed using a transwell chamber (BD Biosciences, USA), and cells were placed in the upper chambers at a density of 5×10^3^ cells/well. Subsequently, medium containing 10% FBS was added to the bottom of the chamber. After incubation for 24 h and fixation with 4% formaldehyde, the migrated cells on the lower surface were stained with 0.1% crystal violet. Finally, the cells were observed and counted using an inverted optical microscope.

### Tumorsphere formation assay

The concentration of GSCs was adjusted to 5×10^3^ cell/mL, and GSCs were cultured in 96-well plates at 100 uL/well with the medium changed every three days. Seven days after seeding, each well was examined, and the number and volume of spheres/cell aggregates were counted.

### 5’-Ethynyl-2’-deoxyuridine (EdU) assay

Cell proliferation was assessed using the EdU assay (Rainbow, Shanghai, China). GSCs were plated in 24-well plates at a density of 5×10^5^ cells/mL. After seven days of cultured, the cells were incubated with 50 μM EdU working solution (5-ethynyl-2’-deoxyuridine preheated to 37°C) for 2 h. The cells were fixed with 4% paraformaldehyde and permeabilized with 0.5% Triton X-100. Afterward, 100 uL of click reaction solution was added to each slide, and nuclei were stained with Hoechst. Finally, the images were obtained using an inverted fluorescence microscope.

### Protein preparation and western blotting

Total protein was prepared from GBM cells using pre-chilled RIPA buffer (Gibco, USA) with a proteinase and phosphatase inhibitor cocktail (Selleck, Shanghai, China). The PVDF membranes were incubated with primary antibodies overnight (**Supplementary Table 2**) at 4°C and then with an HRP-labeled secondary antibody (Zsbio Store-bio, Beijing, China) at room temperature for 1 h. The protein bands were visualized using a chemiluminescence reagent (ECL) kit (Boster, Wuhan, China).

### RNA antisense purification assay

RNA antisense purification (RAP) assay was commissioned by Crystal Energy Biotechnology Co., Ltd. The experimental steps have been reported by Engreitz et al. (31), as follows: assay design, probe design, probe generation, cell harvesting and cross-linking, cell lysis, lysate preparation, hybridization, capture, washing, elution, RNA analysis, and DNA analysis.

### Luciferase reporter assay

HEK293t cells were seeded into 24-well plates and cultured overnight, and let-7e and miR-125a wild-type cells with potential IL6/IL6R binding sites or mutants of each binding site (**Supplementary Table 3**) were generated and fused to the luciferase reporter pGL3-basic plasmid, alone or following pre-treatment with miR-7-5p mimics or inhibitor. According to the manufacturer’s protocol, the relative luciferase activity in each well was analyzed after 24 h using a dual-luciferase reporter assay system (Promega, USA).

### Xenograft model *in vivo*

All animal experiments were approved by the Animal Experiment Administration Committee of the Air Force Military Medical University. All experiments were performed in accordance with the National Research Council’s Guide for the Care and Use of Laboratory Animals. Four-week-old athymic BALB/c nude mice were purchased from the Air Force Medical University Animal Center (Xi’an, China). U87 cells were transfected, and a total of 5 × 10^6^ GBM cells per mouse were stereotactically injected into the skin or brain. The intracranial tumors were measured weekly using bioluminescence imaging. After a month, the subcutaneous tumors were measured. Subsequently, the mice were sacrificed and the tumor tissues were removed for experiments.

### Statistical analysis

All statistical analyses were performed using SPSS (version 19.0; IBM Corporation, USA) and GraphPad Prism9.0 (GraphPad Software Inc., USA). Differences between groups were analyzed using the Student’s t-test or Cox proportional hazards model analysis. The *in vitro* experiments were repeated at least three times, and the data are presented as mean ± standard deviation (SD) from at least three independent experiments. Statistical significance is indicated by *P<0.05, **P<0.01, or ***P<0.001.

## Results

### LncRNA-GAS5 levels are decreased in high-grade glioma tissues and cells

**Figure 1:**
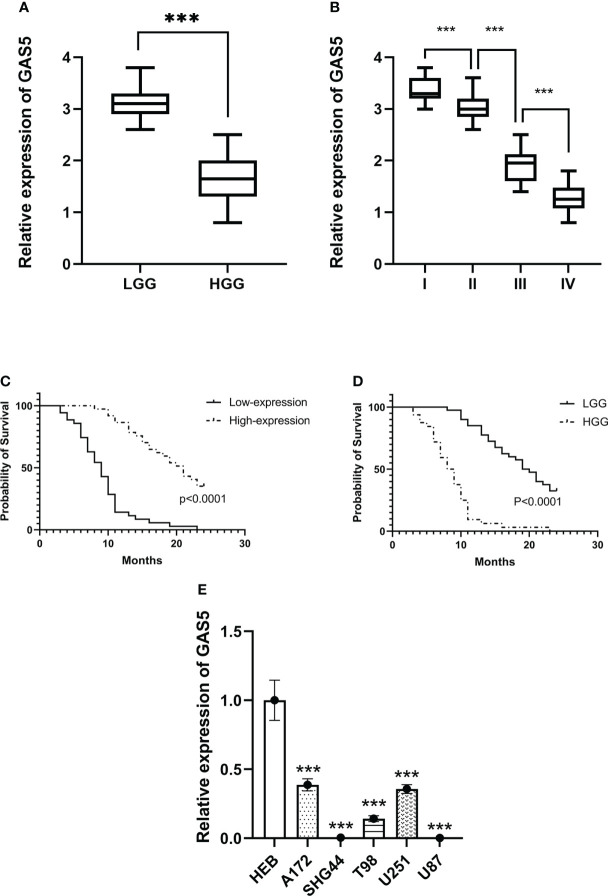
LncRNA-GAS5 is low-expressed in high-grade glioma tissues and cells. **(A) **GAS5 levels are measured in high grade glioma (HGG) tissues (n=36) and low-grade glioma (LGG) tissues (n=36) by qRT-PCR using GAPDH as a reference gene. **(B)** GAS5 levels are measured in WHO I (n=11), II (n=29), III (n=18) and IV (n=14) grade glioma tissues using GAPDH as a reference gene. **(C)** Kaplan-Meier survival curve of patients divided into HGG (n=36) and LGG (n=36) involved in our study is shown. **(D)** Kaplan-Meier survival curve of patients is divided into high-expression group and low-expression group according to the expression of GAS5 involved in our study is shown. **(E)** RNA is extracted from glial cell line (HEB) and five types of glioma cell lines (A172, SHG-44, T98, U251, U87) (n=3 in each cell lines), GAS5 expression level is measured by qRT-PCR using GAPDH as a reference gene. Unless otherwise noted, data are presented as the mean ± S.D. P value is determined by Student’s t-test (***P < 0.001).

Recently, some investigators have shown that lncRNA-GAS5 functions as a protein-binding RNA to directly regulate glioma progression (32, 33). To explore the role of GAS5 in glioma, we determined the expression of GAS5 in 72 glioma samples that were divided into high- and low-grades according to pathological classification. qRT-PCR analysis showed that GAS5 expression was lower in high-grade glioma tissues than in low-grade glioma tissues (**Figure 1A**). Moreover, with an increase in glioma grade, the expression of GAS5 declined drastically (**Figure 1B**), indicating a latent negative relationship between the expression level of GAS5 and glioma grade. To further investigate this correlation, we evaluated the clinical data of 72 patients and found that the pathological grading of glioma and the KPS score of patients significantly correlated with GAS5 expression (**Table 1**). In addition, the median survival of patients with high-grade glioma was significantly lower than that of those with low-grade gliomas (**Figure 1C**). Subsequently, patients were divided into different groups according to GAS5 expression, which revealed that the median survival of the high GAS5 expression group was significantly higher than that of the low expression group (**Figure 1D**). Moreover, we established a Cox proportional hazards model and found that the preoperative KPS score, tumor pathological grade, and lncRNA-GAS5 expression level significantly correlated with postoperative survival time, indicating that they were independent risk factors for patient prognosis (**Table 2**). Furthermore, five types of glioma cells (A172, SHG-44, T98, U251, U87) and one healthy glial cell line (HEB) were used as references. qRT-PCR showed that GAS5 expression considerably decreased in glioma cells (**Figure 1E**). Thus, all data indicated that GAS5 expression decreased in GBM tissues and cells, which might be associated with poor prognosis.

**Table 1:** Characteristics of study population.

Preoperative characteristics	Case (%)	Median survival time ± SD(m)	p value	GAS5/GAPDH(mean ± SD)	P value
Totality	72	13 ± 1.3		1.51 ± 0.50	
Age					
<60 years	58 (80.6%)	13 ± 1.3	0.539	2.43 ± 0.84	0.512
≥60 years	14 (18.4%)	10 ± 0.7		2.59 ± 0.76	
Sex					
Male	40 (55.6%)	11 ± 1.3	0.149	2.43 ± 0.84	0.705
Female	32 (44.4%)	13 ± 2.8		2.50 ± 0.80	
KPS^a^					
100	18 (25%)	16 ± 3.2	0.029*	2.82 ± 0.66	<0.001***
70-90	50 (70%)	11 ± 1.3		2.43 ± 0.79	
<70	4 (5%)	9 ± 2.6		1.13 ± 0.22	
Extent of surgical resection					
Total resection	63 (87.5%)	13 ± 1.2	0.377	2.45 ± 0.83	0.431
Subtotal resection	9 (12.5%)	10 ± 2.2		2.51 ± 0.79	
Tumor pathological type					
Low grade(I, II)	40 (55.6%)	21 ± 2.3	<0.001***	3.11 ± 0.30	0.009**
High grade(III, IV)	32 (44.4%)	9 ± 1.8		1.65 ± 0.44	

Data are expressed as frequency (prevalence in %), mean ± standard deviation, values separated by comma). Statistical analyses are performed using students’ T test.(*P < 0.05, **P < 0.01, ***P < 0.001) ^a^Karnofsky score.

**Table 2:** Reasons for hazard ratio of risk factors.

Risk factors	Hazard ratio(95% CI^a^)	P value
Age	0.86 (0.56-1.41)	0.594
Sex	1.26 (0.81-2.23)	0.337
KPS	1.79 (1.05-3.17)	0.039*
Extent of surgical resection	1.37 (0.89-2.08)	0.446
Tumor pathological type	4.39 (2.76-0.87)	<0.001***
Expression level of GAS5	3.55 (1.96-6.48)	<0.001***

Data are expressed as hazard ratio (95% CI). Statistical analysis is performed using Cox proportional hazards model analysis. (*P < 0.05, ***P < 0.001) ^a^ 95% confidence interval.

### LncRNA-GAS5 repressed the viability and migration of glioma cells

To explore the role of GAS5 in GBM progression, we enhanced or knocked down the expression of GAS5 in U251, U87, A172, T98, and SHG-44 cells by transfecting them with specific GAS5-overexpression or GAS5-shRNA lentiviruses. The results revealed that GAS5 expression remarkably increased in U87, A172, and SHG-44 cells and decreased in U87 cells after transfection (**Supplementary Figure 1A**). Hence, we used these cell lines for follow-up tests.

**Figure 2:**
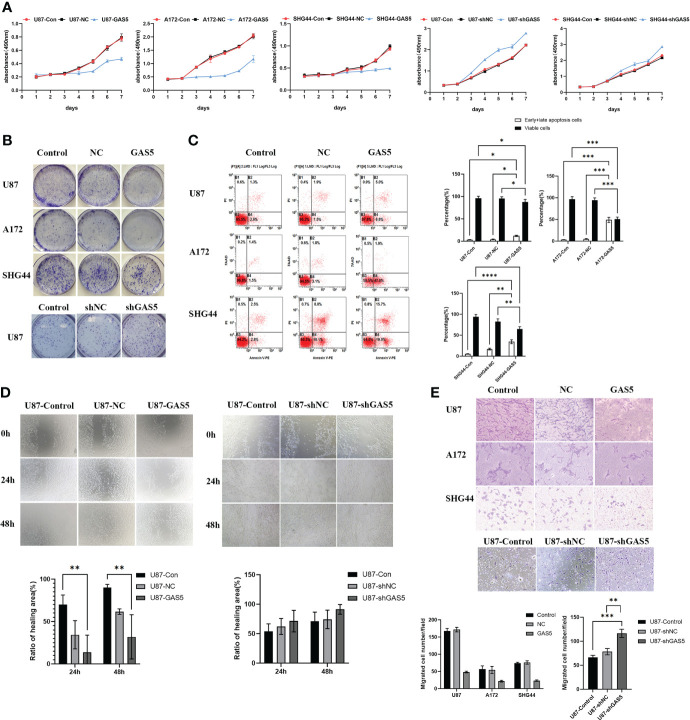
LncRNA-GAS5 overexpression diminished cell viability and suppressed the migration and invasion of GBM cells. **(A)** CCK-8 assay revealed the effect of GAS5 overexpression and knockdwon on the proliferation of parental U87, A172 and SHG44 cell lines with the cells are cultured for seven consecutive days and the absorbance of the cells are measured daily (n=6). **(B)** Colony formation assay of GAS5 overexpression and knockdwon and parental GBM cell lines (n=3). Representative images are shown. **(C)** Flow cytometric analysis of apoptosis in GAS5 overexpression and knockdwon and parental GBM cell lines (n=3). The statistical result of the flow cytometric analysis is shown. **(D)** Scratch test showed the healing ability of U87 cells (n=3). Representative images and the ratio of healing area are shown. **(E)** Transwell assay of the effect of GAS5 on the migration and invasion of GBM cells (n=3). Representative images and the migration cell number are shown. Data are presented as the mean ± S.D. P value is determined by Student’s t test (*P < 0.05, **P < 0.01, ***P < 0.001, ****P<0.0001).

Cell viability was then assayed using the CCK8 assay at various time points (**Figure 2A**). The overexpression of GAS5 drastically diminished cellular survival, whereas knockdown of GAS5 enhanced cell survival. Colony formation assays indicated a significant decline in the rate of colony formation after GAS5 overexpression (**Figure 2B**), and knockdown of GAS5 increased the colony-forming ability of glioma cells, indicating that GAS5 impaired cell self-renewal. As indicated by flow cytometry assays, elevated GAS5 levels suppressed the apoptosis of glioma cells, but knockdown of GAS5 did not have an obvious influence on cell apoptosis (**Figure 2C** and **Supplementary Figure 2C**). In addition, the invasion and migration abilities of GBM cells were assessed. Transwell assays revealed that GAS5 overexpression suppressed the invasive ability of glioma cells (**Figure 2D**). Furthermore, the scratch test indicated that the overexpression of GAS5 inhibited the migration ability of cells (**Figure 2E** and **Supplementary Figure 1B**). Conversely, following GAS5 knockdown, the migratory ability of U87 cells was stronger than that of the control group **(** **Figures 2D**, **E** **)**. Overall, the above experimental results revealed that GAS5 plays a negative role in promoting tumor progression and the malignancy of glioma cells.

### LncRNA-GAS5 overexpression impaired the stemness and proliferation of GSCs

**Figure 3:**
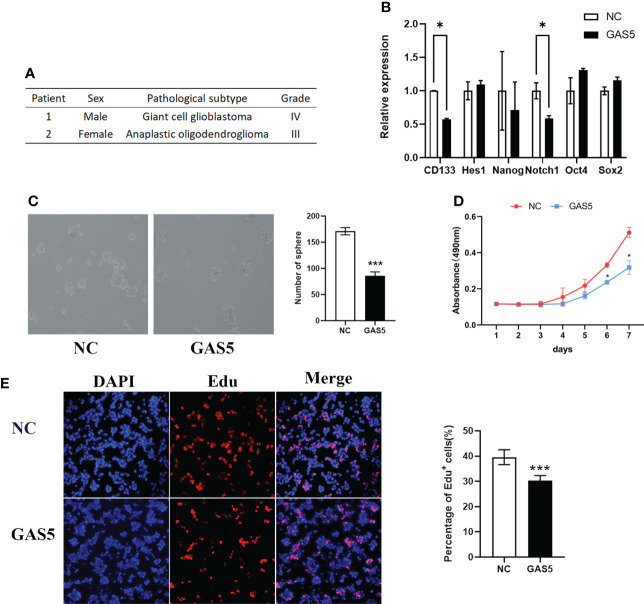
LncRNA-GAS5 over-expression impairs the stemness and proliferation of GSCs. **(A)** Information from the two clinical specimens we used to induce glioma stem cells. **(B)** qRT-PCR analysis of the expression of several stem cell marker molecules in GAS5 overexpression and parental GSCs (n=3). GAPDH is used as a reference gene. **(C)** Tumor spheres formation assay showed the upregulation of GAS5 affected the number and size of tumorspheres (n=3). **(D)** CCK-8 assay revealed the effect of GAS5 overexpression on the proliferation of GSCs with the cells are cultured for seven consecutive days and the absorbance of the cells are measured daily (n=6). **(E)** EdU assay showed the effect of GAS5 overexpression on DNA replication activity of GSC (n = 3). Data are presented as the mean ± S.D. P value is determined by Student’s t test (*P < 0.05, ***P < 0.001).

Although GAS5 has been shown to regulate glioma cells, its detailed mechanisms remain unclear. GSCs play a vital role in GBM progression and tumorigenesis. To clarify the function of GAS5 in GBM stem-like cells (GSCs), we cultured GSCs from GBM surgical specimens and named them GSC-1 and GSC-2 (**Figure 3A**). To further elucidate the stemness of GSC-1 and GSC-2, we analyzed the expression of the GSC marker CD133 and induced cell differentiation (**Supplementary Figures 2A**–**C**). As indicated by the results, GSC-1 and GSC-2 possessed the typical characteristics of stem cells and could be used in subsequent experiments. Further analysis indicated that overexpression of GAS5 in GSCs substantially diminished the expression of stem cell markers, including CD133 and Notch1, which are related to the stemness and malignant progression of gliomas (**Figure 3B** and **Supplementary Figure 2D**). Moreover, the results of tumorsphere formation, CCK-8, and EdU assays further verified the inhibitory role of GAS5 overexpression in GSC proliferation (**Figures 3C–E**). All these data revealed that GAS5 overexpression impaired the stemness and proliferation of GSCs.

### LncRNA-GAS5 restrained the proliferation and migration of GSCs through let-7e and miR-125a by modulating SPACA6

**Figure 4:**
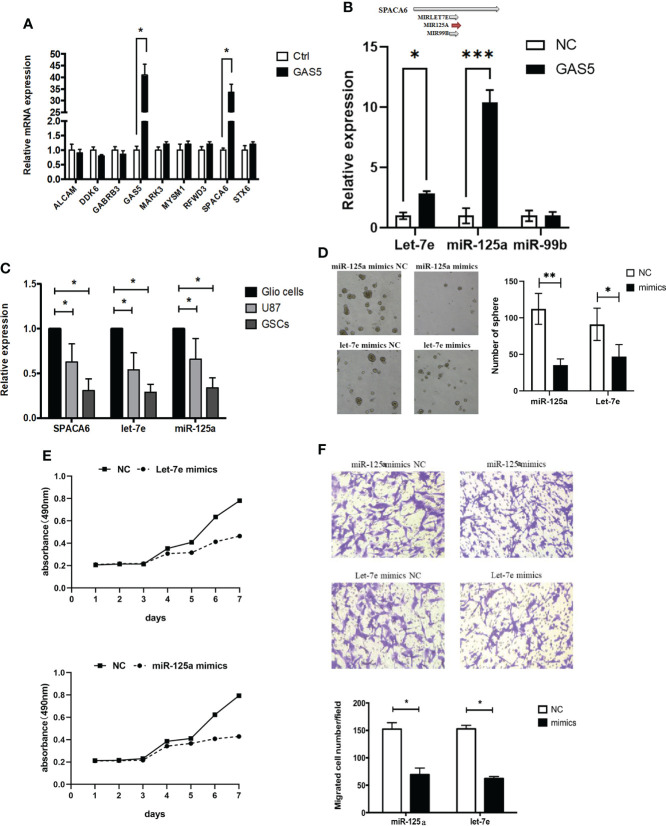
LncRNA-GAS5 restrains the proliferation and migration of GSCs through Let-7e and miR-125a by modulating SPACA6. **(A)** qRT-PCR analysis of the expression of several genes that are significant in the RAP assay in GAS5 overexpression and parental GSCs (n=3). GAPDH is used as a reference gene. **(B)** Upper: Three miRNAs with SPACA6 as host gene (data from https://www.ncbi.nlm.nih.gov/gene/406910). Lower: qRT-PCR analysis of the expression of let-7e, miR-125a and miR-99b in GAS5 overexpression and parental GSCs (n=3). GAPDH is used as a reference gene. **(C)** qRT-PCR analysis of the expression of SPACA6, let-7e and miR-125a respectively in glial cells, GBM cells and GSCs (n=3). GAPDH is used as a reference gene. **(D)** Tumor spheres formation assay showed miR-125a and let-7e mimics affected the number and size of tumorspheres (n=3). **(E)** CCK-8 assay revealed the effect of miR-125a and let-7e mimics on the proliferation of GSCs with the cells are cultured for seven consecutive days and the absorbance of the cells are measured daily (n=6). **(F)** Transwell assay of the effect of miR-125a and let-7e mimics on the migration and invasion of GSCs (n=3). Representative images and the migration cell number are shown. Data are presented as the mean ± S.D. P value is determined by Student’s t test (*P < 0.05, **P < 0.01, ***P < 0.001).

To clarify the molecular mechanism underlying the function of GAS5 in GSCs, we detected mRNAs that interacted with GAS5 in GSCs through an RNA antisense purification (RAP) assay and referred to the enrichment level and function of the gene **(** **Supplementary Table 4** **)**. The results identified *ALCAM*, *DDK6*, *GABRB3*, *MAPK3*, *MYSM1*, *RFWD*, *SPACA6*, and *STX6* as candidate genes that interact with GAS5, which were then subjected to validation by qRT-PCR *via* GAS5-overexpressing GCS. The results indicate that GAS5 substantially promoted SPACA6 expression **(** **Figure 4A** **)**. Considering that SPACA6 is the host gene for let-7e, miR-125a, and miR-99b (https://www.ncbi.nlm.nih.gov/gene/406910), we quantified let-7e, miR-125a, and miR-99b in GAS5-overexpressing GSCs and found that let-7e and miR-125a were upregulated by GAS5 **(** **Figure 4B** **)**. To further detect the correlation between the expression of *SPACA6*, let-7e, miR-125a, and *GAS5*, we evaluated these genes in healthy glial cells, GBM cells, and GSCs. The results indicated that the expression levels of *SPACA6*, let-7e, and miR-125a in GBM cells and GSCs were lower than that in normal glial cells, and the decrease in GSCs was more significant **(** **Figure 4C** **)**. To clarify whether let-7e and miR-125a could act as anti-oncogenes, we transfected GSCs with mimics of these two miRNAs. The stemness properties of GSCs were verified by assessing their ability to form tumorspheres, and the results showed that let-7e and miR-125a mimics inhibited sphere formation **(** **Figure 4D** **)**. Moreover, the CCK-8 assay results showed that let-7e and miR-125a repressed GSCs proliferation **(** **Figure 4E** **)**. A transwell assay was also performed to evaluate the migration of glioma cells, which indicated that let-7e and miR-125a inhibited glioma cell invasion **(** **Figure 4F** **)**. These results showed that let-7e and miR-125a inhibited the stemness, proliferation, and migration of GSCs, which was likely accomplished by the modulation of SPACA6.

### Let-7e and miR-125a suppressed the expression of the IL-6/IL-6R axis

**Figure 5:**
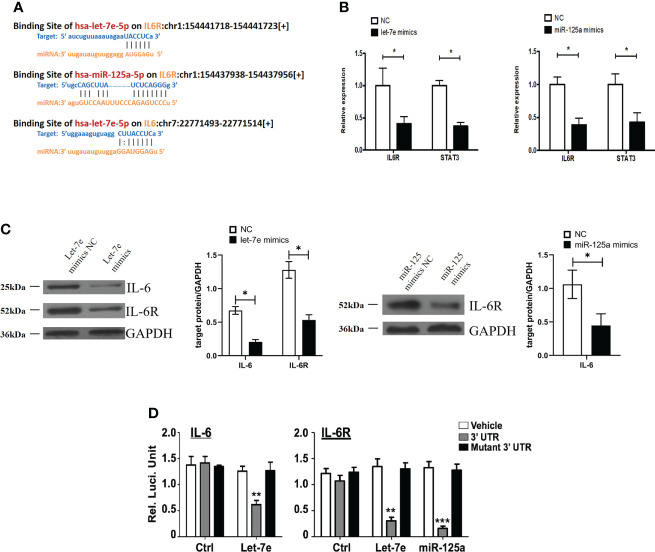
Let-7e inhibited the expression of IL-6 and IL-6R and miR-125a inhibited the expression of IL-6R. **(A)** Diagrams of binding side of let-7e and miR-125a matched the IL6 and IL6R gene. **(B)** Let-7e/miR-125a mimics are co-transfected into the U87 cells respectively, and then the expression of IL-6R is assessed by qRT-PCR analysis (n=3). GAPDH is used as a reference gene. **(C)** Western blot analysis of IL-6 and IL-6R proteins in U87 cells transfected with let-7e and miR-125a mimics (n=3). Quantitative results of western blot analysis are shown. **(D)** Let-7e and miR-125a mimics and reporter plasmids containing the wild type or mutant form of IL-6 3’UTR and IL-6R 3’UTR are co-transfected into the U87 cells, and then the luciferase activity is assessed after 48 h transfection. Expression of IL-6 and IL-6R are shown. Data are presented as the mean ± S.D. P value is determined by Student’s t test (*P < 0.05, **P < 0.01, ***P < 0.001).

Bioinformatics analysis using starBase v2.0 predicted that let-7e and miR-125a shared the same downstream target gene, IL-6R (**Figure 5A**). It has been reported that IL-6 signaling blocks GSC survival through activating STAT3 signaling (34–36). Therefore, we hypothesized that IL-6/IL-6R might play a role in regulating tumor suppressor genes in GBMs cells through a common downstream molecular mechanism. Therefore, we treated U87 cells with mimics of let-7e and miR-125a. The qRT-PCR and western blotting results showed that let-7e inhibited the expression of IL-6 and IL-6R simultaneously, while miR-125a inhibited the expression of IL-6R (**Figures 5B, C**). A luciferase reporter assay was performed to determine whether IL-6 and IL-6R are indeed targets of let-7e and miR-125a, through the 3’ UTR region. The results revealed that upregulation of let-7e resulted in deactivation of the 3’ UTR of IL6 and IL-6R, while the overexpression of miR-125a also decreased 3’ UTR activation of IL-6 (**Figure 5D**). These results indicate that let-7e exerts its biological effect by directly binding to the 3’ UTR of IL6, IL6R, and miR-125a.

### LncRNA-GAS5 plays an antioncogenic role in GBM with the involvement of let-7e and miR-125a

**Figure 6:**
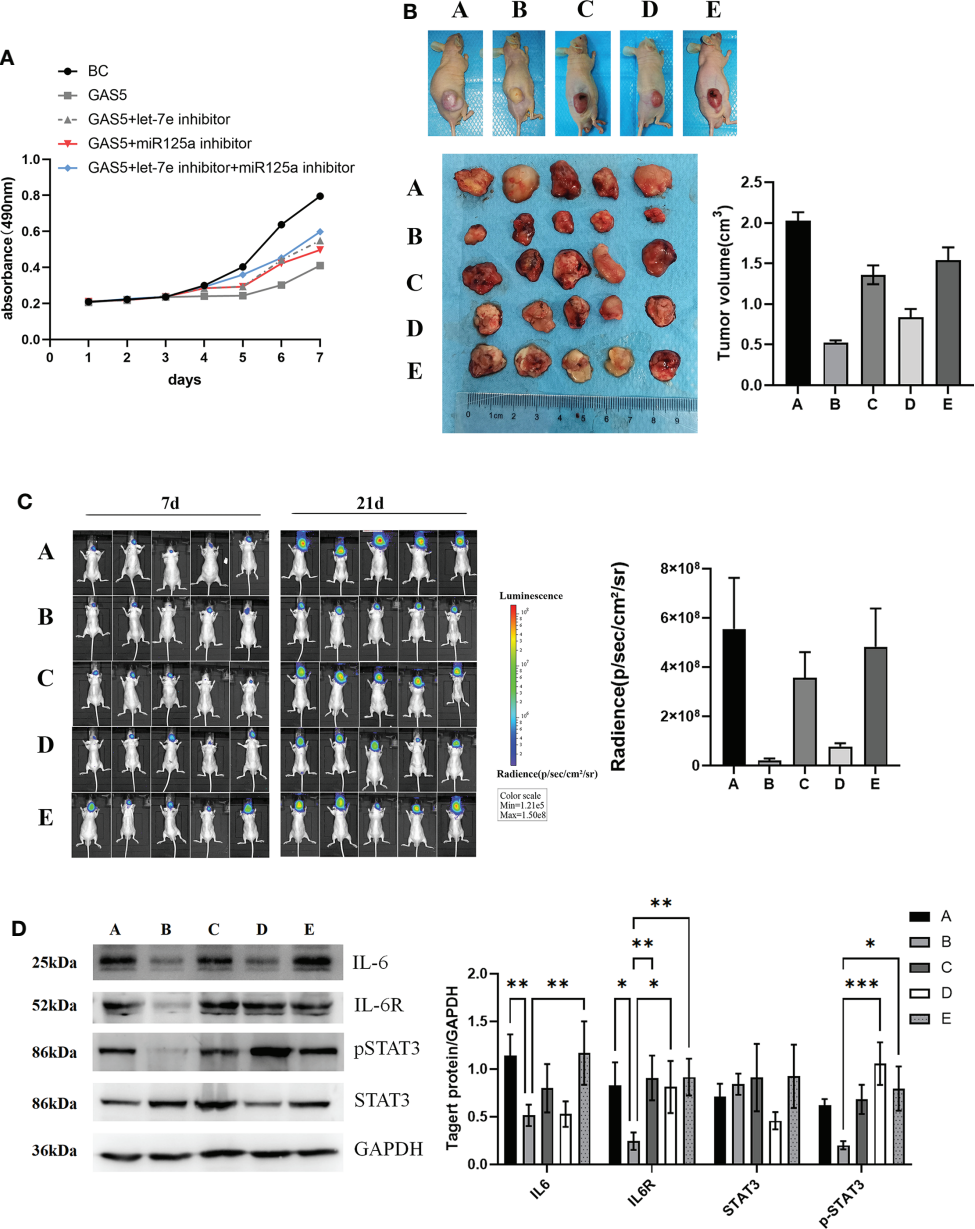
LncRNA-GAS5 plays the role of antioncogene in GBM with the involvement of let-7e and miR-125a. **(A)** Supernatant rescue assay showed the results of CCK-8 assay analysis of the effect of let-7e inhibitor/miR-125a inhibitor transfected on the GAS5 overexpression U87 cells (n=3). **(B)** U87 cells are transfected with GAS5 overexpression lentivirus, miR-125a inhibitor and let-7e inhibitor individually or jointly and they are injected subcutaneously (5×10^6^ cells) into the nude mice (n=5/group). Five images of the subcutaneous tumor of nude mice and the tumor volume are shwon (tumor volume=0.5×l×l×w, l=tumor length, w=tumor width). (a: Control, b: GAS5 overexpression, c: GAS5 overexpression+let-7e inhibitor, d: GAS5 overexpression+miR125a inhibitor, e: GAS5 overexpression+let-7e inhibitor+miR125a inhibitor) **(C)** U87 cells are transfected with GAS5 overexpression lentivirus, miR-125a inhibitor and let-7e inhibitor individually or jointly and they are injected stereotactically into the brain (5×10^6^ cells) into the nude mice (n=5/group). The bioluminescence imaging assay is used to detect the excitation fluorescence in the brain tumor of the nude mice and the relative photons flux is estimated. (a: Control, b: GAS5 overexpression, c: GAS5 overexpression+let-7e inhibitor, d: GAS5 overexpression+miR125a inhibitor, e: GAS5 overexpression+let-7e inhibitor+miR125a inhibitor) **(D)** Proteins are extracted from the nude mice tumors tissues that we formed subcutaneously before. IL-6, IL-6R, STAT3, pSTAT3 are determined by western blot analysis (n=3). Quantitative results of western blot analysis are shown. Data are presented as the mean ± S.D. P value is determined by Student’s t test (*P < 0.05, **P < 0.01, ***P < 0.001).

To investigate the effect of let-7e and miR-125a on GAS5-overexpressing GSCs, a rescue experiment was performed. The results revealed that, after overexpression of GAS5, cellular viability remarkably diminished (**Figure 6A**). However, let-7e and miR-125a inhibitors markedly increased the proliferation of GSCs after GAS5 overexpression (**Figure 6A**). Additionally, when let-7e and miR-125a inhibitors were used simultaneously, the restorative effect on cell viability was stronger (**Figure 6A**). The effects of GAS5, let-7e, and miR-125 on tumor cell growth *in vivo* were verified in nude mice after injection of GAS5 overexpressing U87 cells subcutaneously or stereotactically into the brain. Compared to the control group, the tumorigenic ability of GAS5-overexpressing U87 cells *in vivo* was considerably reduced. Furthermore, the anti-tumor ability of GAS5-overexpression could be inhibited partly by let-7e and miR-125a inhibitors, individually or jointly, and the effect of the let-7e inhibitor was stronger than that of the miR-125a inhibitor (**Figures 6B, C**). To further explore the downstream molecular mechanisms of GAS5, we used tumor specimens from the aforementioned nude mice for validation. The western blotting results showed that the expression of IL6, IL6R, and p-STAT3 could be downregulated by GAS5-overexpression. Moreover, the expression of IL6R and p-STAT3 downregulated by GAS5-overexpression was elevated by let-7e and miR-125a inhibitors, and the expression of IL6 that was downregulated by GAS5 overexpression was elevated by a let-7e inhibitor (**Figure 6D**). These data indicate that lncRNA-GAS5 plays an anti-oncogenic role in GBM *via* the let-7e/miR-125a-IL-6/IL-6R/STAT3 axis.

## Discussion

The latest WHO classification of CNS tumors has updated molecular biomarkers based on tumor morphology to refine the CNS tumor classification (6). ncRNAs are also involved in several cancer-associated processes and are closely related to tumors prognosis (11, 37, 38). GSCs display striking cellular heterogeneity, which is a major contributor to the poor prognosis of patients with glioblastoma (39). Therefore, a better understanding of the molecular mechanisms of GSCs may provide new insights into GBM treatment. The current study revealed that GAS5 suppressed glioma cell growth, both *in vitro* and *in vivo*. We also found that GAS5 positively controls SPACA6, which targets two major microgenes, let-7e and miR-125, to regulate overlapping oncogenic properties in GSCs, of which the inhibition of the downstream IL-6/IL-6R/STAT3 pathway is involved in the molecular mechanism of GAS5 as an antioncogene.

In recent years, the role of GAS5 in tumorigenesis and progression has attracted attention in a wide variety of malignancies, and GAS5 has been discovered to affect various biological processes in tumors. For example, in pancreatic cancer, GAS5 functions as a competing endogenous RNA for miR-221 to suppress cell growth, metastasis, and gemcitabine resistance by mediating epithelial-mesenchymal transformation (EMT) and tumor stem cell self-renewal (40). Liu et al. proved that in glioma, exon 2 of GAS5 directly binds to the miR-18a-5p-binding site to negatively regulate miR-18a-5p, which facilitates the tumor-suppressor functions of GAS5 (41). In this study, we also found that downregulation of GAS5 in glioma could predict poor survival, and GAS5 overexpression could facilitate the migration and invasion of GBM cells and decrease cell viability and the antitumor effect of GAS5 in nude mice. LncRNAs can be secreted extracellularly in the form of exosomes, which makes lncRNAs in body fluids potentially useful as biomarkers to indicate tumor progression and malignancy and guides personalized treatment (25). Our study strongly corroborates the potential of GAS5 as a therapeutic target and clinical predictor of glioma.

Deregulation of ncRNAs has been linked to almost every cancer investigated to date and affects almost all major cancer hallmarks (42, 43). Elaborating the mechanism and crosstalk underlying ncRNAs will provide a deeper understanding of molecular therapeutic strategies and possible mechanisms of glioma pathogenesis. Compared to other types of RNA, lncRNAs have no major molecular prototypes and mainly interact with DNA or RNA in the form of modular domains by nucleic acid base pairing or with proteins *via* advanced RNA structures (44). One of the working mechanisms of lncRNAs that is widely acknowledged is that lncRNAs act as competitive endogenous RNA (competing for endogenous RNA, ceRNA) to competitively bind microRNAs, and the binding site of ceRNAs to mRNA partially relieves the negative regulatory effect of the corresponding miRNA on its target mRNA (45). Emerging evidence has indicated that GAS5 and other lncRNAs could sponge miRNAs to repress their expression and further suppress their downstream pathways, either in glioma or other cancers (32, 40, 41, 46–48). Nevertheless, we found that GAS5 interacts with SPACA6 mRNA and stabilizes its expression to improve the expression levels of let-7e and miR-125a (49). This conclusion could be used to supplement our understanding of how GAS5 acts as a ceRNA in gliomas. LncRNAs could perform their downstream biological functions by combining with mRNA transcripts, and the RAP assay results also suggested that GAS5 and SPACA6 could interact with each other. In addition, our investigation revealed that let-7e and miR-125 individually and synergistically function in tumor suppression in gliomas by blocking their downstream pathways, which adds new evidence to our speculation that GAS5 could protect SPACA6 from degradation by combining with it to act as an anti-oncogene. The finding that let-7e and miR-125a act as suppressors of the IL-6/IL-6R/STAT3 pathway also verified the repression function of the miRNAs in gene expression. In general, the regulatory mechanism proposed in our study enriched and verified the regulatory mechanism of ncRNAs and provided new strategies and targets for ncRNA-targeting glioma therapy.

Glioblastomas contain a specialized subgroup of tumor cells, called GSCs (21). Since the stemness of GSCs plays an essential role in the progression of GBM (7). Zhao et al. found that the GAS5/miR-196a-5p/FOXO1 axis inhibits GSC proliferation, migration, and invasion (48). Our study also revealed that GAS5 overexpression inhibited GSC proliferation, migration, and invasion, and promoted apoptosis by upregulating SPACA6 and its downstream miR-125a/let-7e pathways. Moreover, our study was the first to show that GAS5 can affect the stemness of glioma cells. The stemness of GSCs can be adjusted by epithelial-mesenchymal transition (EMT) (50), growth factor receptor variant III (EGFRvIII) (51), and certain miRNAs, such as miR-205, miR-103a-3p, and miR30 (52–54). It has also been reported that IL-6 signaling disturbs GSC survival through STAT3 activation (35). Cortez et al. suggested that miR-29b and miR-125a are the potential regulators of invasion in glioblastoma. Hence, we clarified that let-7e inhibited the expression of IL-6 and IL-6R simultaneously, and miR-125a inhibited the expression of IL-6R. LncRNA-GAS5 acts as an antioncogene in GSCs *via* the IL-6/IL-6R/STAT3 pathway. The let-7 gene is an essential developmental gene and let-7-family members many function as tumor suppressors in various cancers (55). Let-7e was previously identified to be downregulated by nuclear paraspeckle assembly transcript 1 (NEAT1) to promote oncogenesis in GSCs (56). In this study, however, we found that let-7e could inhibit GSC progression, which might relate different upstream and downstream miRNA regulatory crosstalk mechanisms. As a member of the lin-4 family of microRNAs, miR-125a plays a fundamental role in cell differentiation and development (57), and the reduction of the malignant progression of GSCs by downregulated miR-125a has not yet been reported. Our findings complement the mechanism by which ncRNAs act in the formation and progression of GSCs. However, to fully reveal the subcellular localization of GAS5 in glioma cells and further define the underlying mechanism by which GAS5 interacts directly with proteins, additional evaluations are warranted.

## Conclusion

In summary, our study verified that the under-expression of GAS5 was related to a higher degree of malignancy and shorter survival time after surgery. GAS5 negatively regulates the malignant behavior of glioma cells and GSCs. Notably, GAS5 upregulated the expression of let-7e and miR-125a by reducing SPACA6 degradation and further repressing activation of the downstream IL6/IL-6R/STAT3 pathway. However, the complete elucidation of the underlying mechanism requires more evidence, and it will be important to determine whether GAS5 can directly bind to SPACA6 to prevent its degradation and affect downstream pathways.

## Data Availability Statement

The original contributions presented in the study are included in the article/**Supplementary Material**. Further inquiries can be directed to the corresponding authors.

## Ethics Statement

The studies involving human participants were reviewed and approved by Research Ethics Committee of Xijing Hospital of Airforce Medical University. The patients/participants provided their written informed consent to participate in this study. The animal study was reviewed and approved by Animal Experiment Administration Committee of the Air Force Military Medical University.

## Author contributions

SZL, ZF and JLZ designed research and wrote the manuscript, SW, KXR, JZ, JL, BJ, XQW, YND, TTW and WHL performed experiments, XWF, YH and XH analyzed data, LW, YGW and ZF provided clinical samples, SZL and SW interpreted results of experiments and write the manuscript. All authors reviewed and approved the manuscript.

## Funding

This study was supported by grants from National Natural Science Foundation of Shaanxi (2017JM8073).

## Conflict of interest

The authors declare that the research was conducted in the absence of any commercial or financial relationships that could be construed as a potential conflict of interest.

## Publisher’s note

All claims expressed in this article are solely those of the authors and do not necessarily represent those of their affiliated organizations, or those of the publisher, the editors and the reviewers. Any product that may be evaluated in this article, or claim that may be made by its manufacturer, is not guaranteed or endorsed by the publisher.
