# Supplementary material for: LncRNA GAS5 represses stemness and malignancy of gliomas via elevating the SPACA6-miR-125a/let-7e Axis
Source: Front Oncol. 2022 Aug 29;12:803652. doi: 10.3389/fonc.2022.803652 (PMC9465381; doi:10.3389/fonc.2022.803652)
Supplement: Supplementary file 1 [file DataSheet_1.pdf]

Supplementary Material

A

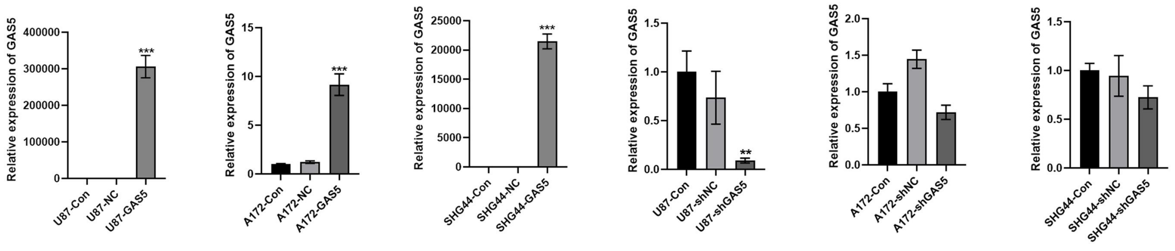

B

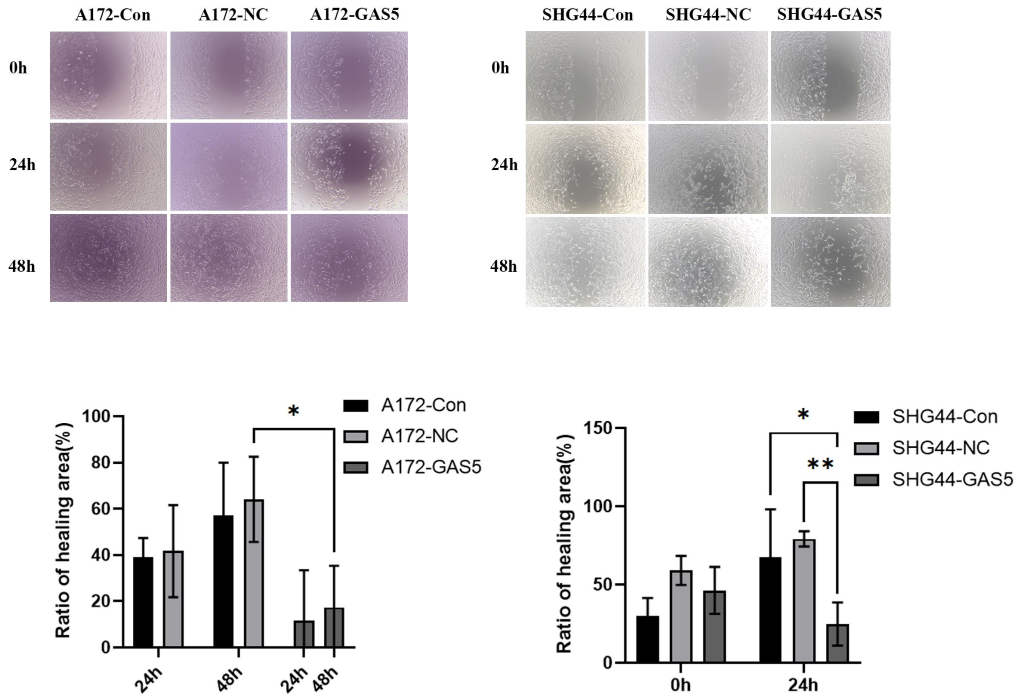

C

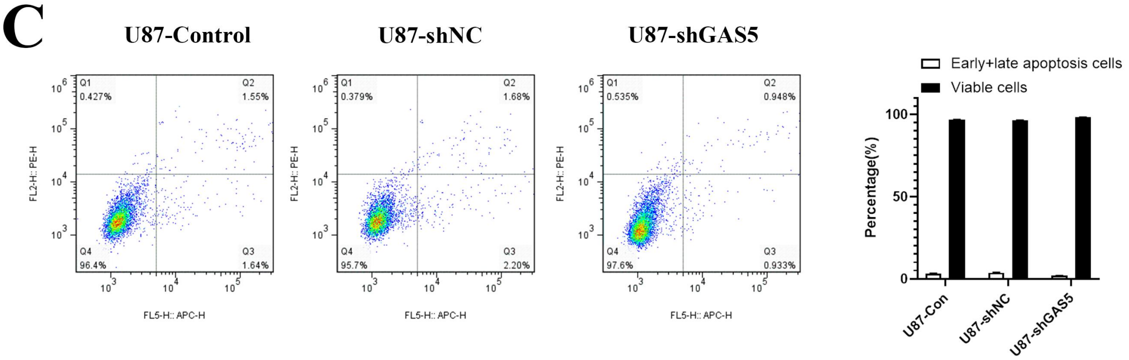

Supplementary Figure 1

(A) We transfect U87, A172 and SHG44 cells with GAS5-overexpressionlentivirusor GAS5-shRNA lentivirus. Transfection efficiency is verified by the measurement of GAS5 expression level by qRT-PCR using GAPDH as a reference gene (n=3).
(B) Scratch test show the healing ability of A172 andSHG44 glioma cells (n=3). Representative images and the ratio of healing area are shown(n=3).
(C) Flow cytometric analysis of apoptosis in GAS5 knockdown and parental U87 cell lines (n=3).

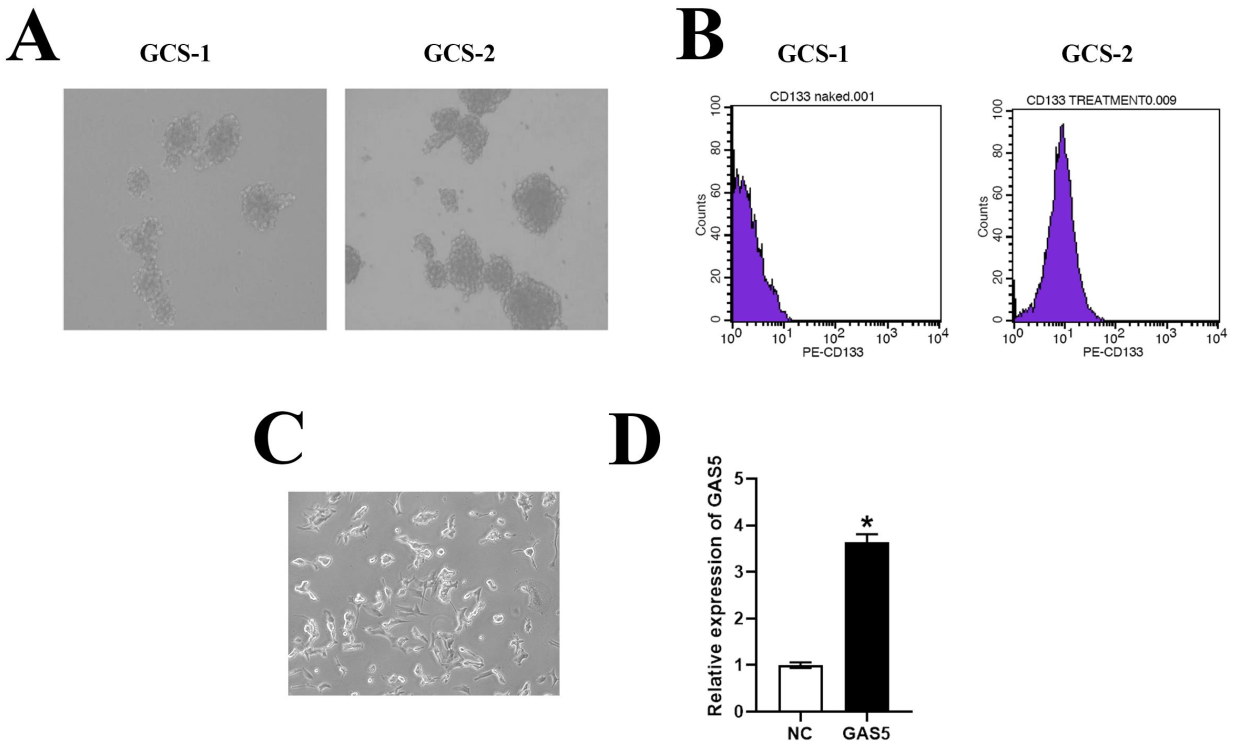

Supplementary Figure 2

(A) The images of cells extracted from patients.
(B) The ratio of cells single-labeled CD133 is assessed by flow cytometry in GSCs-1 and GSCs-2.
(C) The GSCs are induced into differentiation.
(D) We transfect GSCs with GAS5 lentivirus. Transfection efficiency is verified by the measurement of GAS5 expression level by qRT-PCR using GAPDH as a reference gene（n=3).

Supplementary Table 1: Sequences of primers used for qRT-PCR

**Gene**	**Forward (5'-3')**	**Reverse (5'-3')**
lnc-GAS5	CAACTTGCCTGGACCAGCTT	TCAAGCCGACTCTCCATACC
CD133	AGTGGCATCGTGCAAACCTG	CTCCGAATCCATTCGACGATAGTA
Hes1	TGGAAATGACAGTGAAGCACCTC	TCGTTCATGCACTCGCTGAAG
Notch1	TGGGTGCACTCTTGGCATACA	CACGCGGATTAATTTGCATCTG
SOX2	ACACCAATCCCATCCACACT	GCAAACTTCCTGCAAAGCTC
Nanog	CAGATGCAAGAACTCTCCA	GTAGGAAGAGTAAAGGCTG
Oct4	GTGGAGAGCAACTCCGAT	TGCAGAGCTTTGATGTCCTG
ALCAM	ATTATCATACCTTGCCGACTTG	TGTATTCTGGTACATCGTCGTACTG
CDK6	GTGACCAGCAGCGGACAAATAA	AGCAAGACTTCGGGTGCTCTGTA
GABRB3	GCAGAACTGCACTCTGGAAATTGA	TCCACTCCGGTAACAGCCTTG
MARK3	GTAACACAGCATCTGGTGGAATGAC	TGATACTGTGTGTTGAAGCAACTGG
STX6	GAATGCACGCCAGAGCTGAA	AAGTTGTTGGCTACCAGACCCATC
RFWD3	CAGTGTAGGGCCAATGAGAACAA	TGGGTCCTGCTCACCTGAAAG
MYSM1	GGCAGAAGTGATTGGTCTGTTAGGA	GAGGCCTGTGTTTGTGATACAGGA
SPACA6	GGCGACCAGGCTATGTTTTC	GGCATATCTCGGAAATAGGACAAG

GAPDH	GCACCGTCAAGGCTGAGAAC	TGGTGAAGACGCCGTGGA
miR-125a	TCCCTGAGACCCTTTAACCTGTGA	
let-7e	TGAGGTAGGAGGTTGTATAGTT	
miR-99	CACCCGTAGAACCGACCTTGCG	

Supplementary Table 2: Primary antibodies used in the present study

**Antigens**	**Manufacturer**	**Catalogue numbers**	**Application**
IL-6	Invitrogen	# M620	1μg/ml for WB
IL-6R	Invitrogen	# PA5-100836	1:1000 for WB.
STAT3	GeneTex	GTX104616	1:1000 for WB.
p-STAT3	GeneTex	GTX118000	1:1000 for WB.

Supplementary Table 3: Sequences of predicted and mutant binding sites used for luciferase reporter assay

**Name**	**Sequence**(5’-3’)
miR-125a-IL6R-S	TCGAGAAGATGCTTCTCACTGCCATGCCAGCTTATCTCAGGGGTGTGCGGCCTTTGGGC
miR-125a-IL6R-AS	GGCCGCCCAAAGGCCGCACACCCCTGAGATAAGCTGGCATGGCAGTGAGAAGCATCTTC
let-7e-IL6R-S	TCGAGGATGGAGATCACATCTGTTAAATAGAATACCTCAACTCTACGTTGTTTTCTTGC
let-7e-IL6R-AS	GGCCGCAAGAAAACAACGTAGAGTTGAGGTATTCTATTTAACAGATGTGATCTCCATCC
let-7c-IL6-S	TCGAGCCAGATCATTTCTTGGAAAGTGTAGGCTTACCTCAAATAAATGGCT

	AACTTAGC
let-7c-IL6-AS	GGCCGCTAAGTTAGCCATTTATTTGAGGTAAGCCTACACTTTCCAAGAAATGATCTGGC
miR-125b-IL6R-Mut-S	TCGAGAAGATGCTTCTCACTGCCATGCCAGCTTATCACTGCGGTGTGCGGCCTTTGGGC
miR-125b-IL6R-Mut-AS	GGCCGCCCAAAGGCCGCACACCGCAGTGATAAGCTGGCATGGCAGTGAGAAGCATCTTC
let-7c-IL6R-Mut-S	TCGAGGATGGAGATCACATCTGTTAAATAGAATTCGTGAACTCTACGTTGTTTTCTTGC
let-7c-IL6R-Mut-AS	GGCCGCAAGAAAACAACGTAGAGTTCACGAATTCTATTTAACAGATGTGATCTCCATCC
let-7c-IL6-Mut-S	TCGAGCCAGATCATTTCTTGGAAAGTGTAGGCTTTCGTGAAATAAATGGCTAACTTAGC
let-7c-IL6-Mut-AS	GGCCGCTAAGTTAGCCATTTATTTCACGAAAGCCTACACTTTCCAAGAAATGATCTGGC

Supplementary Table 4: Representative results of RAP assay.

The contents of Supplementary Table 4 was separately displayed in the EXCEL file named 'Supplementary Table 4 Representative Results of RAP Assay.xlsx' of supplementary files.
